# Quantitative Readability Assessment of the Internal Medicine Online Patient Information on Annals.org

**DOI:** 10.7759/cureus.4184

**Published:** 2019-03-06

**Authors:** Ahmad A Abu-Heija, Maya Shatta, Mustafa Ajam, Usama Abu-Heija, Nashat Imran, Diane Levine

**Affiliations:** 1 Internal Medicine, Wayne State University, Detroit Medical Center, Detroit, USA; 2 Miscellaneous, Jordan University of Science and Technology, Amman, JOR; 3 Nephrology, Wayne State University, Detroit Medical Center, Detroit, USA

**Keywords:** comprehension, readability, health education, patient education, health resources

## Abstract

Background

Approximately 90% of Americans have access to the internet with the majority of people searching online for medical information pertaining to their health, or the health of loved ones. The public relies immensely on online health information to make decisions related to their healthcare. The American Medical Association (AMA) and the National Institute of Health (NIH) recommend that publicly available health-related information be written at the level of the sixth-seventh grade.

Materials and methods

Patient education materials available to the public on the Annals.org, a website sponsored by the American College of Physicians, were collected. All 89 patient education articles were downloaded from the website and analyzed for their ease of readability. The articles were analyzed utilizing a readability software generating five quantitative readability scores: Flesch Reading Ease (FRE), Flesch-Kincaid Grade Level (FKGL), Gunning Fog Index (GFI), Coleman-Liau Index (CLI), Simple Measure of Gobbledygook (SMOG). All scores, with the exception of FRE, generate a grade level that correlates with the required school-grade level to ensure adequate readability of the information.

Results

Eighty-nine articles were analyzed generating an average score as follows: FRE 62.8, FKGL 7.0, GFI 8.6, CLI 9.6 and SMOG 9.8. Overall, 87.6% of the articles were written at a level higher than the 7th-grade level, which is recommended by the AMA and NIH.

Conclusion

In an era of increased reliance on the internet for medical information pertaining to patients’ health, materials written at a higher grade than recommended has the potential to negatively impact patients’ well-being, in addition to tremendous ramifications on the healthcare system. Potentially redrafting, these articles can prove beneficial to patients who rely on these resources for making healthcare-related decisions.

## Introduction

In an era of widespread internet availability, it is estimated that approximately 90% of Americans have access to the internet, with more than 80% of internet users searching online for medical information. This translates to more than eight million Americans searching the internet for health-related information, on any given day [1-2]. Only a third of these users discuss this health information with their healthcare providers [1]. Moreover, 53% of health seekers report that the information obtained had an impact on how they take care of themselves or someone else [1]. This is in part because of the ease by which information could be found online and the fact that more than three-quarters of Americans own a smartphone and have access to the internet at the tip of their fingers [3].

Unfortunately, the 2003 National Assessment of Adult Literacy (NAAL) showed that 14% of Americans could not read, or understand text written in English and were only able to comprehend very basic, simple text [4]. Furthermore, almost half of Americans lack sufficient literacy required to appropriately comprehend and implement medical treatment and preventive health care, with grave economic consequences [4-6]. According to the NAAL report, uninsured adults have lower health literacy than insured adults [4]. In addition, limited health literacy is prevalent and associated with lower socioeconomic status, comorbidities, and poor health care access, which suggests that limited health literacy can be considered an independent risk factor for the disparities in health faced by the older population. Studies have shown that adults older than 65 years with lower health literacy were more likely to utilize the emergency department and experience higher costs during those visits [7].

Therefore, it is of utmost importance to define health literacy and eHealth literacy in this era. The Institute of Medicine defines health literacy as “the degree to which individuals have the capacity to obtain, process, and understand basic health information and services needed to make appropriate health decision” [8]. Multiple other definitions are proposed by various other entities, with varying degrees of emphasis on eHealth literacy, where eHealth literacy is defined as “a set of skills and knowledge that are essential for productive interactions with technology-based health tools” [9]. One of the crucial qualities of health literacy is the readability of health literature that is directed towards laypeople, with readability defined as “the ease with which written materials are read” [10]. Readability is paramount, as increased readability correlates with increased comprehension [10]. Multiple readability assessment tools are in place to assist authors in addressing the public with materials that are comprehensible and more effortlessly understood by readers. Yet, online patient information is often written at a level that is beyond the comprehensibility of the majority of the population [6,11-13]. Internal medicine ailments encompass a wide variety of illnesses, especially in the older generations. Hence, the requirement of easily readable materials to facilitate understanding and potentially improve adherence and outcomes. 

In an effort to address the extent of inadequacies related to health literacy, the US Department of Health and Human Services (USDHHS), the American Medical Association, and the National Institute of Health have all published guidelines related to the readability of patient-related information (e.g. hand-outs, consent forms, health education materials). These organizations recommend that health information directed towards patient be written at the level of sixth- to seventh-grade reading level, corresponding to a reading level associated with ages 11-13 years [5,10,14]. Per the USDHHS, a sixth-grade reading level is categorized as “easy to read”, while any material between the seventh- and the ninth-grade level is categorized as “average difficulty” and any literature written beyond that level as “difficult” [10]. The purpose of this study was to evaluate the readability of patient education materials that are available on the “Annals of Internal Medicine: Patient Information” website, with measures taken to assess the difficulty of the information written and the complexity by which the articles were formulated.

## Materials and methods

Online patient education materials available to the public from “Annals of Internal Medicine: Patient Information” website, http://annals.org/aim/pages/patient-information [15], were retrieved in September 2018. This website, sponsored by the American College of Physicians (ACP), offers healthcare-related information in the form of brief summaries of studies and clinical guidelines published in the Annals of Internal Medicine journal, targeting patients and interested lay people.

A total of 93 hyperlinks were found on the website, with one duplicate hyperlink for information related to colon cancer screening and three hyperlinks yielded unavailable pages. Duplicate and unavailable links were excluded from analysis. Articles that were targeting physicians or practitioners were excluded. The hyperlinks were all patient-related information. The text from the 89 remaining articles was copied and pasted as plain text into individual documents using Microsoft® Word® (Microsoft, Redmond, Washington, USA). The text was reviewed by the authors independently. During the review, all medical terms followed by explanation were removed from the text prior to analysis, for example when mentioned “sputum; mucus brought up with coughing”, the word sputum would be removed from the text. Likewise, when a medical procedure is explained, for example, when “endoscopic retrograde cholangiopancreatography, which examines the pancreas through a tube inserted down the throat into the stomach and pancreas”, the words endoscopic retrograde cholangiopancreatography were removed. Medical terms or procedures not explained in the text were not removed. In addition, all hyperlinks, tables, advertisements, figures, images, and tables were removed. Further editing was done on the remaining text with expunction of any headings, bullet points, and decimals. References were also expunged, as well as author names and websites. Names of medications, whether brand or generic, were similarly removed from the text.

The articles were then analyzed for their readability levels using the readability website https://readable.com. Five validated scales were then used to quantitatively analyze the articles (Table 1). The scales used were: Flesch Reading Ease (FRE), Flesch-Kincaid Grade Level (FKGL), Gunning Fog Index (GFI), Coleman-Liau Index (CLI), Simple Measure of Gobbledygook (SMOG) [16-20]. The SMOG scale is the preferred assessment of healthcare literature [21]. All the scales generate a readability grade that correlates with a typical school grade level, with 0-12 being from kindergarten to 12th grade and scores higher than 12 correlate with their college degree hierarchical equivalent. The only exception is FRE, which generates a score out of 100, with scores 0-30: very difficult to read, written at the levels of college graduates, 50-60: fairly difficult to read, written at the level of 10th to 12th grade, 60-70: plain English, written at the level of 8th to 9th grade, 70-80: fairly easy to read, written at the level of 7th grade, and 90-100: very easy to read, easily understood by an average 11-year-old student.

**Table 1:** Readability scales used to analyze the articles from patient education materials on Annals of Internal Medicine website

Readability Scale	Variables	Formula
Flesch Reading Ease (FRE)	Average number of syllables (B), average number of words per sentence (W), average number of sentences (S)	206.835 – (84.6 x (B/W)) – (1.015 x (W/S))
Flesch Kincaid Grade Level (FKGL)	Average number of syllables per word (SY) and the average number of words per sentence (W)	(0.39 x W) + (11.8 x SY) – 15.59
Gunning Fog Index (GFI)	Number of sentences (S), number of words (W), number of words with three or more syllables (C)	0.4 x (W/S + ((C/W) x 100))
Coleman-Liau Index (CLI)	Average number of letters per 100 words (L) and the average number of sentences per 100 words (S)	(0.0588 x L) – (0.296 x S) – 15.8
Simple Measure of Gobbledygook (SMOG)	Average number of words with 3 or more syllables (C) and the average number of sentences (S)	1.043 x √ (C x (30/S)) + 3.1291

## Results

The readability grades for the 89 articles obtained from Annal.org online patient information were analyzed. Each article was analyzed individually using five readability scales. The average readability score using the FRE for the collective articles was 62.8, indicating a level of readability that is of average difficulty, per the USDHHS, which is higher than the recommended readability level by the USDHHS, NIH, and AMA [5,10,14]. Table 2 presents the readability scores for the 89 individual articles, in addition to the final column displaying the average grade level for each article.

**Table 2:** List of articles with the generated FRE, FKGL, GFI, CLI and SMOG indices; last column is the average grade level obtained FRE: Flesch Reading Ease, FGKL: Flesch Kincaid Grade Level, GFI: Gunning Fog Index, CLI: Coleman Liau Index, SMOG: Simple Measure of Gobbledygook

Topic	Flesch Reading Ease	Flesch Kincaid Grade Level	Gunning Fog Index	Coleman Liau Index	SMOG Index	Average Grade Level
Abdominal Aortic Ultrasound	77.9	5.2	6.8	5.7	8	6.4
Acne	78.9	5.2	6.8	6.2	7.6	6.5
Acute Colonic Diverticulitis	58.7	7.2	7.6	9.6	9	8.4
Acute Gastrointestinal Bleeding	52.1	9.1	10.8	12.5	11.4	11.0
Acute Kidney Injury	65.9	5.8	7.3	7.7	8.7	7.4
Acute Pancreatitis	53.3	9.1	11	11.8	11.2	10.8
Acute Sinusitis	49.7	9.6	11	11	11.4	10.8
Alcohol Use	57.2	6.9	8	9.4	10.1	8.6
Aortic Stenosis	67.4	6.2	8.4	8.5	9.5	8.2
Asthma	69.9	6.8	8.6	9.7	9.2	8.6
Atopic Dermatitis (Eczema)	58.8	7.6	9.4	11.1	10.4	9.6
Atrial Fibrillation	79	4.7	6.8	6.9	8.4	6.7
Breast Cancer Screening and Prevention	74.2	5.3	7.2	8.9	8.8	7.6
Care of the Adult Cancer Survivor	48.6	9.5	11	13.1	11.3	11.8
Care of Returning Military Personnel	34.5	10.3	11.1	14.3	11.4	11.2
Carpal Tunnel Syndrome	69.9	6.1	7.4	9.1	8.5	7.8
Celiac Disease	49.4	10	12.2	12	12.1	11.6
Chlamydia and Gonorrhea Infection	39.7	10.2	12.4	13.2	12	12.0
Chronic Kidney Disease	67.4	6	6.4	9.6	8.1	7.5
Chronic Obstructive Pulmonary Disease	61.1	7.2	9.3	10.8	9.9	9.3
Clostridium difficile Infection	50.9	9.5	12.2	10.9	12.1	11.2
Common Cutaneous Parasites	67.2	6.7	8.5	9	9.3	8.4
Community-Acquired Pneumonia	65	6.7	8.7	8.4	9.9	8.4
Concussion	66.1	6.5	9	8.3	10	8.5
Constipation	62.9	6.6	7.6	9.9	9	8.3
Contraception	46.1	9.3	12	13.7	12	11.8
Deep Venous Thrombosis	83.8	4.1	5.9	6.5	7.7	6.1
Delirium	29.1	11.1	13	13.4	11.6	12.3
Dementia	57	7.7	9.6	10.9	10.8	9.8
Depression	67.7	5.9	8.6	8.2	9.6	8.1
Diabetic Ketoacidosis	66.8	7.6	10	8.2	10.5	9.1
Dyslipidemia	70.1	5.5	8	7.5	9.1	7.5
Eating Disorders	37.8	9.9	12	12.8	11.3	11.5
Epilepsy	62.5	6.7	8.7	9.3	9.8	8.6
Gastroesophageal Reflux Disease	62.6	7	7.8	10.4	9.4	8.7
Generalized Anxiety Disorder	30.2	11	13.2	15	12	12.8
Gout	78.2	4.7	6.5	7.6	7.9	6.7
Hearing Loss	67.1	6.4	8.5	8.8	9.5	8.3
Heart Failure	61.4	6.7	8.8	10.6	10	9.0
Heart Failure with Preserved Ejection Fraction	76	4.9	7.6	8.3	9	7.5
Hepatitis C Virus	67.4	7	9.3	7.5	10.2	8.5
Herpes Zoster	75.4	4.7	6.4	6.6	8	6.4
Hip Fracture	55.2	8.5	11.1	10.9	11.2	10.4
Hypertension	67.3	6.4	7.9	10.2	9.1	8.4
Hyperthyroidism	41	9.9	10.7	13.6	11	11.3
Hyponatremia	62.1	7.3	7.8	8.6	9.5	8.3
Hypothyroidism	35.7	10.4	11.2	14	10.8	11.6
Influenza	76.2	4.9	6.7	7.1	8.7	6.9
Insomnia	61.7	6.7	7.8	9.9	9.2	8.4
Irritable Bowel Syndrome	63.6	6.3	7.8	8.5	9.2	8.0
Low Back Pain	83.3	3.8	5.5	5.7	7.2	5.6
Lyme Disease	75.3	5.6	7	7.7	8.3	7.2
Management of Newly Diagnosed HIV Infection	69	6.5	8.6	8.5	9.6	8.3
Menopause	62.1	7.5	9.9	10.6	10.7	9.7
Migraine	68.4	6.1	7.9	9.6	9.2	8.2
Multiple Sclerosis	55.8	8.5	10.4	12.1	11.1	10.5
Nephrolithiasis	89.4	3.5	5.3	5	6.4	5.1
Obesity	78.5	5.1	7.9	6.8	9	7.2
Obstructive Sleep Apnea	57.5	8.1	9.4	11.3	10.2	9.8
Osteoarthritis	69.2	5.8	8.5	8.4	9.4	8.0
Osteoporosis	68.3	5.8	7.3	7.9	8.9	7.5
Palliative Care	73.1	5.2	7.3	8.1	8.8	7.4
Peripheral Arterial Disease	89.6	2.9	5.2	5.4	6.9	10.5
Perimenopause	50.1	8.3	10.9	11.6	11	5.1
Pharyngitis	44.7	10.3	12.4	13.5	12.5	12.2
Plantar Fasciitis	57.5	7.4	9.7	9.9	9.9	9.2
The Polycystic Ovary Syndrome	61.2	7.4	9.8	10	10.4	7.2
Polymyalgia Rheumatica	72.5	5.5	6.6	8.1	8.5	10.1
Preoperative Evaluation	64.1	8.1	11.3	9.4	11.5	7.4
Prostate Cancer	71.5	5.7	7	8.4	8.5	9.9
Pulmonary Hypertension	60.6	7.5	10.6	10.6	10.8	7.3
Restless Legs Syndrome	72	5.4	6.7	8.7	8.5	10.3
Rheumatoid Arthritis	55	8.3	10.2	11.9	10.8	8.2
Rotator Cuff Disease	67.5	6.5	7.7	9	9.5	9.3
Sarcoidosis	56.5	7.6	9	10.5	10.1	9.7
Screening for Colorectal Cancer	59.3	8.4	9.5	10.5	10.3	10.3
Sickle Cell Disease	54.2	8.7	10.2	11.1	11	7.7
Smoking Cessation	70.8	5.4	7.7	8.7	8.9	11.5
Stable Ischemic Heart Disease	51.1	9.4	12.1	12.4	11.9	7.4
Substance Use Disorders	76.4	5.2	7.6	7.6	9.3	7.8
Systemic Lupus Erythematosus	66.6	6.3	6.8	9.4	8.6	9.4
Transient Ischemic Attack	70.1	5.9	7.3	9.3	8.8	7.8
Transitions of Care	42.5	10.5	12.9	14.6	12.6	12.7
Travel Medicine	76.9	5.1	7.8	6.7	9	7.2
Tuberculosis	59.7	7.1	8.5	9.3	9.4	8.6
Type 2 Diabetes	71.4	5.5	6.9	8.1	8.8	7.3
Urinary Tract Infection	66.4	6.1	7.4	7	9	7.4
Vaginitis and Cervicitis	42.1	10.1	12.3	11.6	12.3	11.6
Venous Leg Ulcers	81.1	4.3	6.7	6.3	8.4	6.4

**Figure 1:**
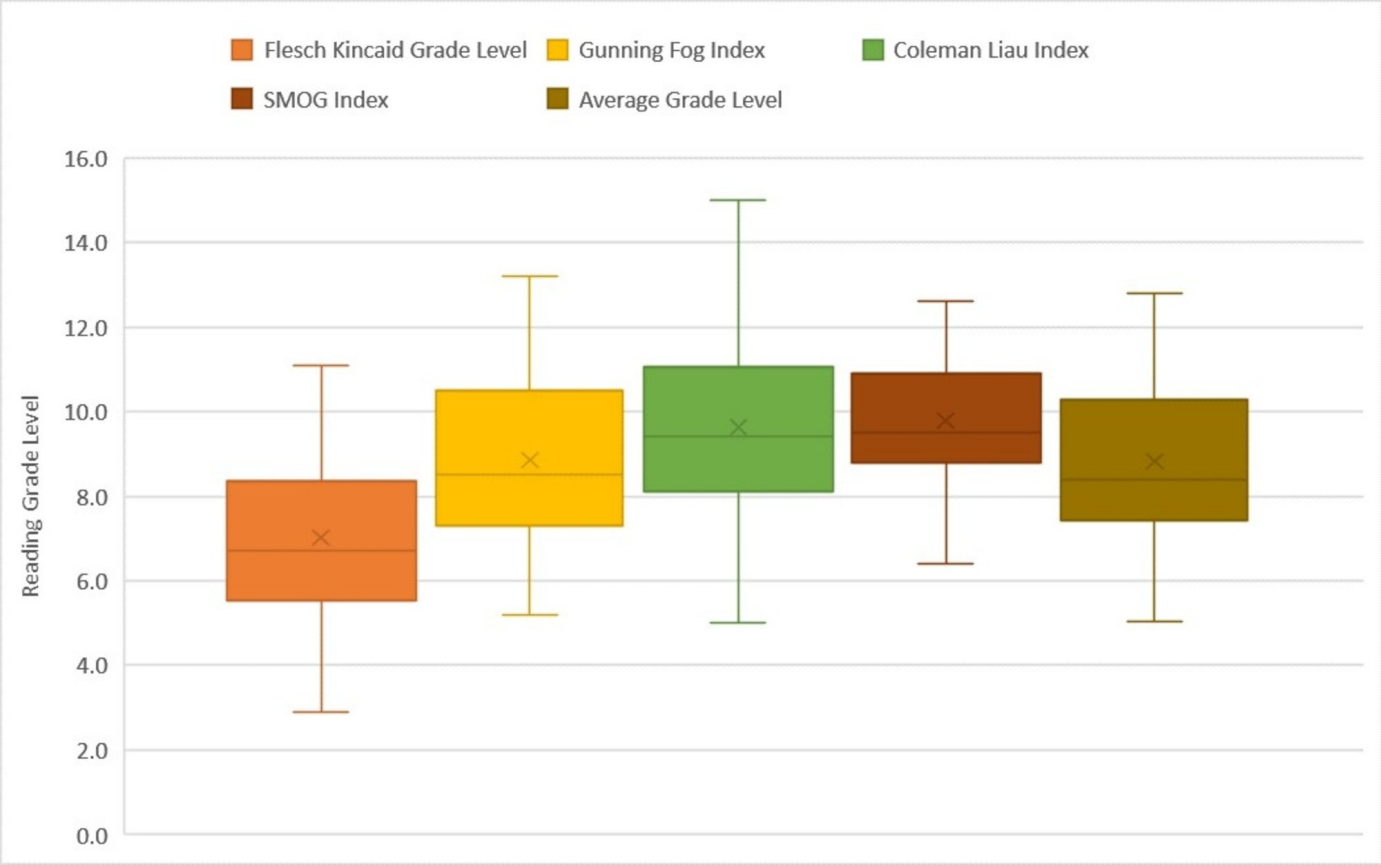
Box and whisker plot displaying reading grade level distribution using all tests, except FRE for all topics reviewed The mean for the articles using FKGL is 7.0; the whiskers range from 2.9 to 11.1. The mean for the articles using GFI is 8.9; the whiskers range from 5.2 to 13.2. The mean for the articles using CLI is 9.6; the whiskers range from 5.0 to 15.0. The mean for the articles using the SMOG index is 9.8; the whiskers range from 6.4 to 12.6. The mean for all the articles using an average grade level combining the former is 8.8; the whiskers range from 5.1 to 12.8. SMOG: Simple Measure of Gobbledygook

**Figure 2:**
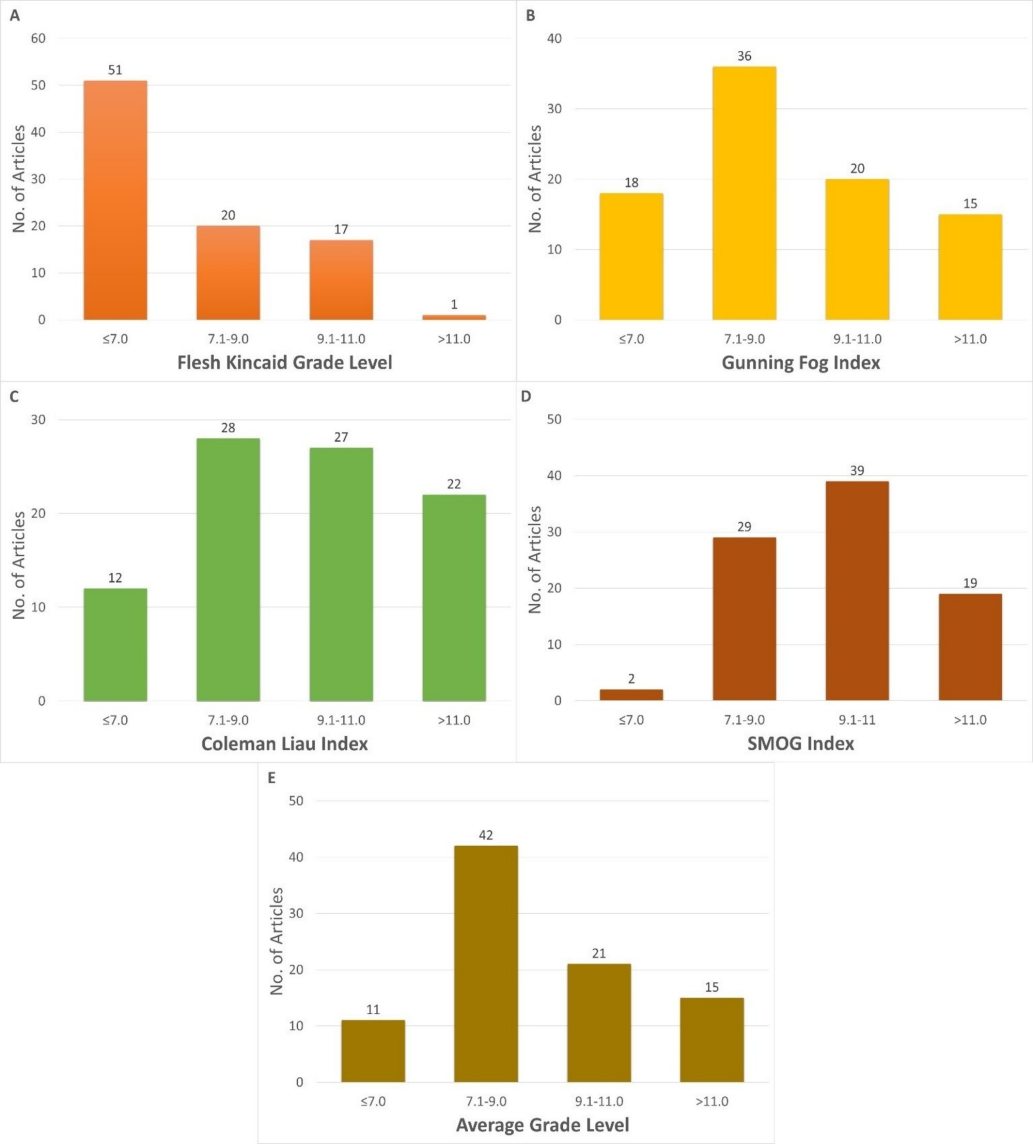
Distribution of readability grades of all the analyzed articles using the five readability indices SMOG: Simple Measure of Gobbledygook

The FRE score for the articles had an average value of 62.8, with a range of scores between 29.1 and 89.6, with less than a third of the articles attaining a score more than 70.0, which translates to articles easily understood by seventh-grade level. Utilizing the FKGL score, the average readability grade level of the collective articles was 7.0, with a range between 2.9 and 8.2. The GFI scale produced an average reading level of 8.6, with scores ranging between 5.2 to 13.2, with more than 80% of the articles written above the recommended readability grade level. In addition, both the CLI and SMOG showed higher average readability scores respective averages of 9.6 and 9.8, as seen in Figure 1. The maximum score obtained on the CLI score was 15, with more 77 of the articles written above the recommended level. Furthermore, SMOG analysis showed minimum readability of 6.4 and a maximum of 12.6, with only two articles written at or below the recommended readability level. However, the SMOG score intrinsically yields higher scores because of a 100% comprehension goal during analysis, yet it has been recommended for healthcare literature with a comprehensibility correlation of 0.88 [20-21]. The number of articles written at each readability grade level, comparing the different scores utilized is shown in Figures 2A-2E.

## Discussion

Patient reliance on online education materials to enhance their well-being and determine when to visit a physician have increased exponentially over the past decade, with more than 50% of internet users reporting that information found on the internet affected their decision in treating a medical condition [1]. While online education materials found on the patient information site on Annals.org provide patients with a valuable, evidence-based resource for maintaining their well-being, readability of the content of these materials is variable, with 87.6% of articles written above the seventh-grade level.

Health literacy is complicated, with multiple variables that play part in the overall comprehensibility of educational materials [22]. Individuals’ level of education and familiarity with medical terminology are fundamental in assessing the level of understanding they will attain in reading online education materials. Health literacy is heavily dependent on the education attained by patients, with lower health literacy rates linked to poorer health outcomes, including increased hospitalizations, poorer health status and higher mortality [23]. Studies have shown lower mammography studies and lower influenza immunizations among patients with lower health literacy [23]. On a larger scale, lower health literacy rates are associated with the tremendous economic cost to the US economy, with estimates ranging between $70 and $230 billion US dollars annually [5-6].

Comparing the readability of online education materials found on Annal.org to other major medical societies shows better overall readability of the materials with an average grade level being 8.8 ± 1.8 (SD) [6,10-13]. Using readability scales to assess comprehensibility of education materials carries inherent flaws and has its own limitations since essentially all the algorithms used, take into account word length and the number of syllables in the words to assess readability. For example, more difficult medical words such as “lipid” or “ketone”, which are short in length can be interpreted as more readable than longer words, such as “hospitalization”, which is longer, yet more understandable by the general population. Nonetheless, it remains true that the materials obtained are written beyond the recommended readability of the American population. Revisions to the materials can effectively increase readability, increasing comprehension among readers with limited health literacy capabilities. Utilizing graphics and videos can also increase the comprehension of complex health information that is difficult to clarify using textual information. Attaining a goal of near-universal comprehension can improve health outcomes and potentially minimize the financial burden associated with these outcomes.

## Conclusions

The online patient education materials found on the Annals.org website provide patients with an excellent source of information to enable them to care for themselves and loved ones. Nevertheless, the majority of the materials are written at a readability level higher than recommended by the AMA and NIH. Given the integral role that online patient education materials play in the decision-making process of patients and follow-up care with physicians, greater emphasis should be placed on the readability and comprehensibility of online educational materials. 
